# Insight to shape of soil microbiome during the ternary cropping system of *Gastradia elata*

**DOI:** 10.1186/s12866-020-01790-y

**Published:** 2020-05-05

**Authors:** Qing-Song Yuan, Jiao Xu, Weike Jiang, Xiaohong Ou, Hui Wang, Lanping Guo, Chenghong Xiao, Yanhong Wang, Xiao Wang, Chuanzhi Kang, Tao Zhou

**Affiliations:** 1 Guizhou University of Traditional Chinese Medicine, Dongqingnan Road, Guiyang, 540025 Guizhou China; 2 National Resource Center for Chinese Materia Medica, China Academy of Chinese Medical Sciences, Beijing, 100700 China; 3 Shandong Analysis and Test Center, Shandong Academic of Sciences, Jinan, 250014 Shandong China

**Keywords:** *Gastradia elata*, *Armillaria mella*, Microbiome, Rhizosphere soil, Mycorrhizosphere soil, Ternary cropping system, Next generation pyrosequencing

## Abstract

**Background:**

The ternary cropping system of *Gastradia elata* depends on a symbiotic relationship with the mycorrhizal fungi *Armillaria mellea*, which decays wood to assimilate nutrition for the growth of *G. elata*. The composition of microbe flora as key determinants of rhizoshere and mycorrhizoshere soil fertility and health was investigated to understand how *G. elata* and *A. mellea* impacted on its composition. The next generation pyrosequencing analysis was applied to assess the shift of structure of microbial community in rhizoshere of *G. elata* and mycorrhizoshere of *A. mellea* compared to the control sample under agriculture process.

**Results:**

The root-associated microbe floras were significantly impacted by rhizocompartments (including rhizoshere and mycorrhizoshere) and agriculture process. Cropping process of *G. elata* enhanced the richness and diversity of the microbial community in rhizoshere and mycorrhizoshere soil. Furthermore, planting process of *G. elata* significantly reduced the abundance of phyla Basidiomycota, Firmicutes and Actinobacteria, while increased the abundance of phyla Ascomycota, Chloroflexi, Proteobacteria, Planctomycetes, and Gemmatimonadetes in rhizoshere and mycorrhizoshere. Besides, *A. mellea* and *G. elata* significantly enriched several members of saprophytoic and pathogenic fungus (i.e., *Exophiala*, *Leptodontidium*, *Cosmospora*, *Cercophora*, *Metarhizium*, *Ilyonectria*, and *Sporothrix*), which will enhance the possibility of *G. elata* disease incidence. At the same time, the ternary cropping system significantly deterred several members of beneficial ectomycorrhizal fungus (i.e., *Russula*, *Sebacina*, and *Amanita*), which will reduce the ability to protect *G. elata* from diseases.

**Conclusions:**

In the ternary cropping system of *G. elata*, *A. mellea* and *G. elata* lead to imbalance of microbial community in rhizoshere and mycorrhizoshere soil, suggested that further studies on maintaining the balance of microbial community in *A. mellea* mycorrhizosphere and *G. elata* rhizosphere soil under field conditions may provide a promising avenue for high yield and high quality *G. elata*.

## Background

Rhizosphere soil is an important compartment direct contact with plants root or mycorrhiza rhizomorph, which play critica roles to assimilate nutrients, resist stress, and combat disease. Furthermore, rhizosphere microbes are key determinants of soil fertility and health. Imbalance of soil microbes have been identified as the most important factor contributing to elevated disease incidence in plants [1, 2]. For examples, many researchers reported that the imbalance of microbial community in soil is the main reason for consecutive monoculture problem of many medicinal plants, such as *Pseudostellaria heterophylla* [3, 4], *Rehmannia glutinous* [5, 6]. What’s more, soil microbes have great influence on soil biogeochemical processes including decomposition, mineralization, and retention of soil nutrients [7]. Conversely, plants also can significantly affect the composition of soil microorganisms, change soil physical and chemical properties during agricultural operations through secret phytochemicals [8]. For example, Haichar el al’s researches demonstrated that plant-derived phytochemicals secreted by root are able to structure the rhizosphere microbiome by deterring or attracting certain microbial species [9]. And also *Radix notoginseng* eventually caused a shift in soil microbes from a bacterial-dominant community to a fungal-dominant community through increase N application rates in its agricultural ecosystems [10].

Microbial community structure in soil is very complicated, in which the microbes in soil commonly includes bacteria, fungi, protozoa, etc. Alternation of microbial composition, especially the structure change between bacteria and fungi,can make plants susceptible to diseases and poor growth. The main reason of the altenation of microbial composition in soil is the interactions between bacteria, fungi, such as the antagonistic effect of probiotics and pathogenic fungi, which plays crucial roles in maintaining the balance of microbial community in soil [11, 12]. To date, in tuberous medicinal plant, numerous studies have shown that plant root rot complex are main caused by the increase of the abundance of pathogenic fungus including *Fusarium* spp. [13, 14], *Rhizoctonia solani* [15], *Alternaria* spp. [16, 17], *Phytophthora sojae* [18] in soil. Many biocontrol researches managing crops disease demonstrated that antagonism bacteria, for example *Bacillus amyloliquefaciens* S76–3 as soil amendment and improvement, have a good application to manage the disease of various crops [19–21]. Additionally, Wu et al. pointed out that the relative abundances of *Fusarium*, *Cylindrocarpon*, *Rhizoctonia*, etc. remarkably increased after agriculture process of *Rehmannia glutinosa*, as well the relative abundance of beneficial bacterial *Pseudomonas aeruginosa* decreased after agriculture process of *Rehmannia glutinosa* [5]. Therefore, It is very important for *G. elata* production to determine the alternation of bacteria and fungi community in its ternary cropping system.

As a perennial herbaceous plant, *G. elata* belongs to the achlorophyllous orchid and is widely distributed in Asian. *G. elata* is highly valued in medicine, health care and food, primarily due to the various active compounds of *G. elata*, such as gastrodin, polysaccharide, organic acid, etc. [22–24]. The main producing areas of *G. elata* mainly contain the eastern Asian countries such as Chinese, Southern Korea, Japanese, etc. Especially in china, the cultivation of *G. elata* is the most common. The artificial cultivation system of *G. elata* is very special, in which the mycorrhizal fungi *A. mellea* decomposes litter and trees, assimilates nutrition to promote the growth of *G. elata* [25, 26]. And more, previously studies showed that a number of crops could reshape the microbiome of rhizosphere soil under agriculture process, such as potato [27], soybean [1], wheat [2], and banana [28], as well as Chinese medicine herbs such as *Pseudostellaria heterophylla* [3, 4], *Rehmannia glutinous* [5, 6]. But at present, there is no report on whether *G. elata* and *A. mellea* have impact on the community structure and the abundance of the microflora in rhizosphere or mycorrhizoshere soil under agriculture process.

Besides, early studies have dominated that *A. mellea,* as a medicine fungi could produce many antibacterial and antifungal compounds, such as sesquiterpene aryl esters and armillaric acid, which exhibits high inhibitory activity against gram-positive bacteria, yeast, *Streptococcus* spp., *Mucor* spp., *Trichoderma* spp., *Rifai* aggr., *Rhizopus stoloniferp*, *Fusarium* spp., and *Gliocladium viren* [29–32]. What’s more, *A. mellea* possess strongly antifungal activity because it have the ability to induce *G. elata* to produce a set of defense proteins [33, 34]. But, it is not clear how *G. elata* and *A. mellea* affect the microbial community in the rhizosphere soil in the ternary cropping system. Therefore, we conducted *G. elata* planting experiments on the *G. elata* geo-authentic producing area Dafang County in China. Then, we collected the (mycor) rhizsphere soils after agriculture process and used the next generation sequencing technology of ITS and V3V4 rDNA amplicons in Illumina MiSeq platform to characterize microbial communities in rhizosphere and mycorrhizosphere soils and assess how *G. elata* and *A. mellea* shape the soil microbial community structure after *G. elata* cropping process.

## Results

### General statistic of reads and OTUs in sequencing data

**Fig. 1:**
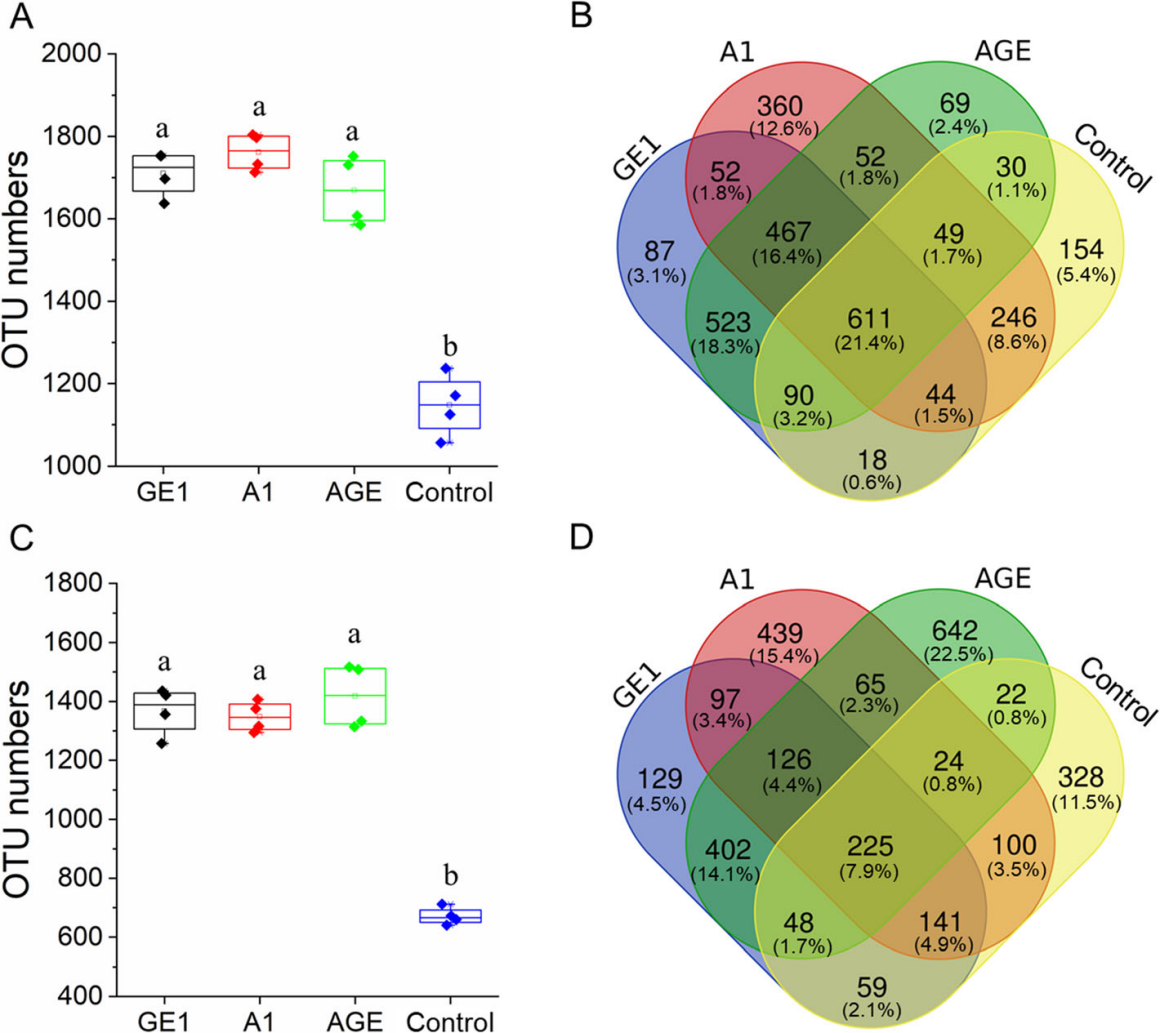
OTU numbers and venn analysis exclusive and shared OTUs in different rhizoshere and mycorrhizoshere soil samples. **A** the numbers of OTU bacterial community; **B** the venn diagram in bacterial community; **C** the numbers of OTU fungal community; **D** the venn diagram in fungal community. GE1, A1, AGE, and control represent the rhizoshere or mycorrhizoshere soil of *G. elata* tubers, *A. mellea* rhizomorphs, *G. elata* tubers with *A. mellea* rhizomorphs, and unplanted, respectively. Lowercases a and b in figures show significantly differences between the different rhizocompartment soils (GE1, A1, AGE and control), determined by Tukey’s test (*p* < 0.05)

The next generation sequencing (NGS) of V3V4 and ITS rDNA amplicons was applied to assess the changes in the rhizosphere and mycorrhizosphere microbial communities after *G. elata* cropping process. After removal of short and low-quality reads, singletons, and chimeras, 19,555 from 34,971 effective sequences were obtained for V3V4 rDNA amplicons, as well as 48,999 from 67,295 effective sequences were obtained for ITS rDNA amplicons in total, and the detailed information of sequencing data in each sample was displayed in supplementary Table S1 and S2. Based on 97% similarity, 1710, 1762, 1669,and 1148 OUTs were obtained for bacterial community in the rhizoshere soil samples from the mature tube of *G. elata* (GE1), rhizomorph of *A. mellea* (A1), the mother tube of *G. elata* containing rhizomorph of *A. mellea* (GEA) and no planted (control), respectively. As well as based on 97% similarity 1367, 1348, 1418, and 671 OTUs were obtained for fungal community in GE1, A1, GEA, and control, respectively (Fig. 1).

### Alpha diversity analysis in different Rhizoshere soils

**Fig. 2:**
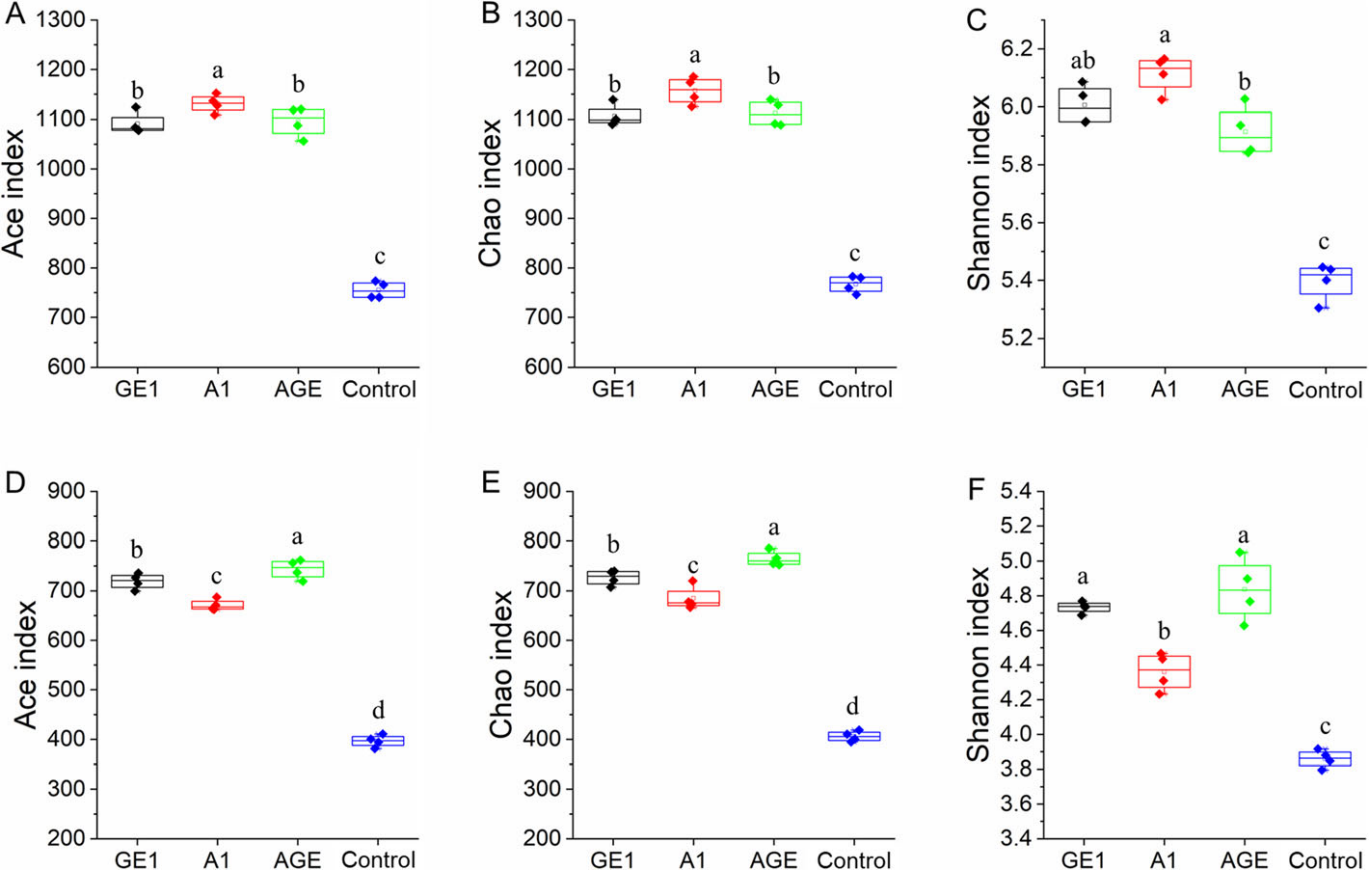
Diversity indices in different rhizoshere and mycorrhizoshere soil samples. **A**, **B**, and **C** represent the Ace, Chao, and Shannon indices of bacterial community, respectively; **D**, **E**, and **F** represent the Ace, Chao, and Shannon indices of fungal community, respectively. GE1, A1, AGE, and control represent the rhizoshere or mycorrhizoshere soil of *G. elata* tubers, *A. mellea* rhizomorphs, *G. elata* tubers with *A. mellea* rhizomorphs, and unplanted, respectively. Lowercases a, b, c and d in figures show significantly differences between the different rhizocompartment soils (GE1, A1, AGE and control), determined by Tukey’s test (*p* < 0.05)

The microbiota diversity and richness of different rhizoshpere soil samples were investigated by Ace, Chao, Shannon indexes based on ITS and V3V4 sequence data. In bacteria community, A1 showed the highest diversity and highest species richness with the observed species, Chao, Ace indices at 1762, 1131.2, 1157.2, respectively (Fig. 2). Control had the lowest species richness and diversity compared with the other three rhizosphere soil groups (Fig. 2). All indices of richness and diversity demonstrated AGE and GE were significantly different from the control, but no significant difference was observed between AGE and GE. In fungi community, the richness and diversity of A1 were lower than AGE and GE, but higher than control (Fig. 2 D, E, F). Similarly, the simposon indices had the same results (Fig. S1). The results showed that *G. elata* and *A. mellea* could elevate the richness and diversity in rhizoshere and mycorrhizoshere soil, and *A. mellea* had the lower effect on the fungi community than the bacteria community in mycorrhizoshere soil.

### Microbiota diversity in different Rhizoshere soils under *G. elata* cropping process

**Fig. 3:**
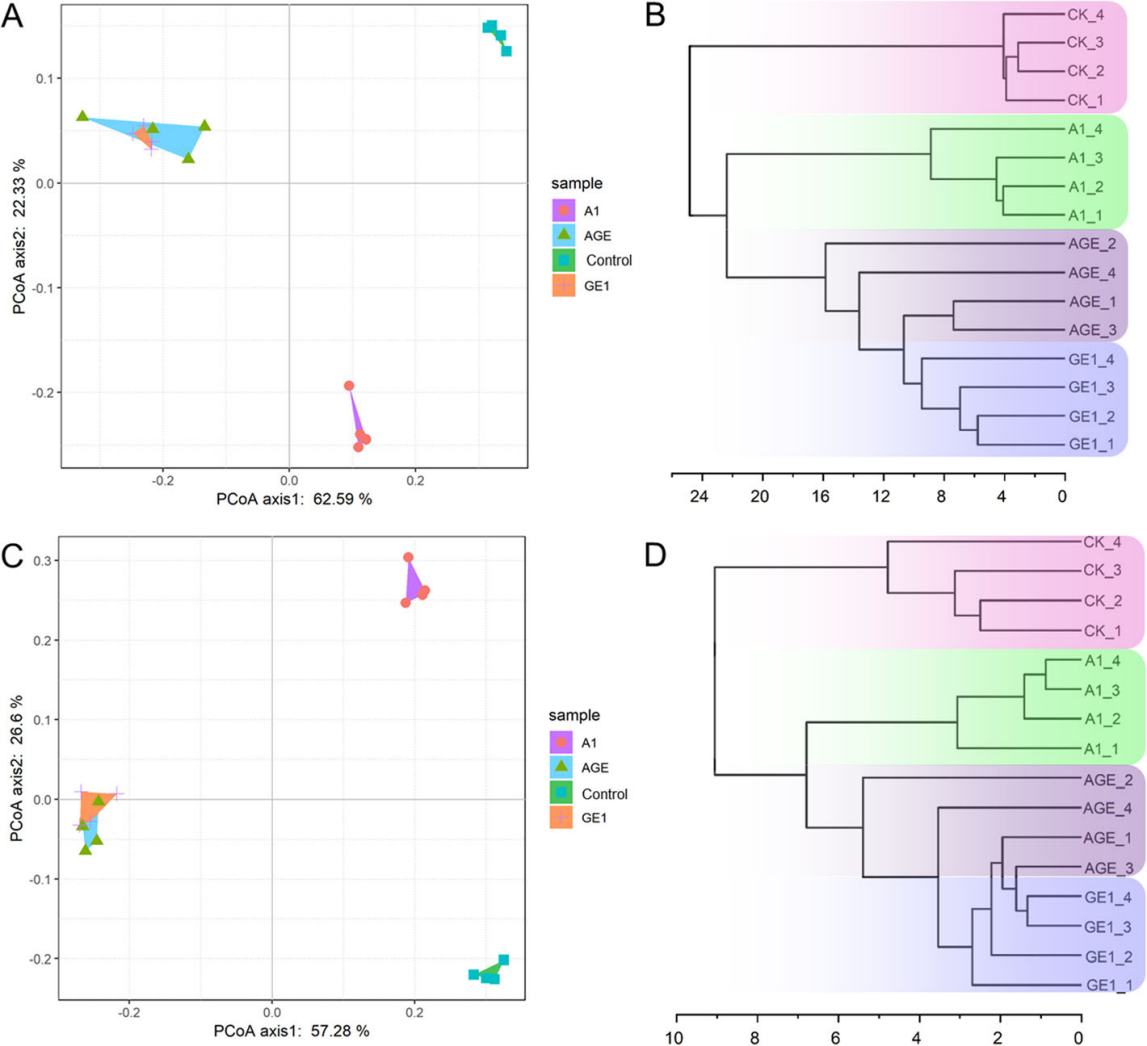
Principal coordinate analysis (PCoA) based on weighted UniFrac metric (WUF) and Hierarchical clustering analysis (HCA) based on the Bray-Curtis distance (BCD). **a** and **b** represent the PCoA analysis and HCA analysis of bacterial community, respectively; **c** and **d** represent the PCoA analysis and HCA analysis of fungal community, respectively. GE1, A1, AGE, and control represent the rhizoshere or mycorrhizoshere soil of *G. elata* tubers, *A. mellea* rhizomorphs, *G. elata* tubers with *A. mellea* rhizomorphs, and unplanted, respectively

In order to investigate the alteration patterns of microbiota in the different rhizoshere soils, the beta diversity patterns were estimated by the principal coordinate analysis (PCoA) and Hierarchical clustering analysis (HCA) based on the weighted UniFrac metric (WUF) algorithm and the Bray-Curtis distance (BCD) algorithm, respectively (Fig. 3). PCoA indicated that the microbiota community was separated by the sites of rhizoshere in *G. elata* cropping process, apart from GE1 and AGE. The first two axes PCoA1 and PCoA2 accounted for 62.59 and 22.33% of total variation in V3V4 (Fig. 3a), as well as 57.28 and 26.6% of total variation in ITS (Fig. 3c), respectively. And HCA clustering also had the similar results (Fig. 3b, d). Furthermore, venn diagram analysis showed that the numbers of peculiar OTU in A1, AGE, GE1, and control groups were much higher than that of shared OTU in paired comparison analysis. But the numbers of shared OTU in AGE and GE1 showed no significant difference from the numbers of peculiar OTU in AGE or GE1 (Fig. 1B, D).

### Alternation of microbiota community structure under *G. elata* cropping process

**Fig. 4:**
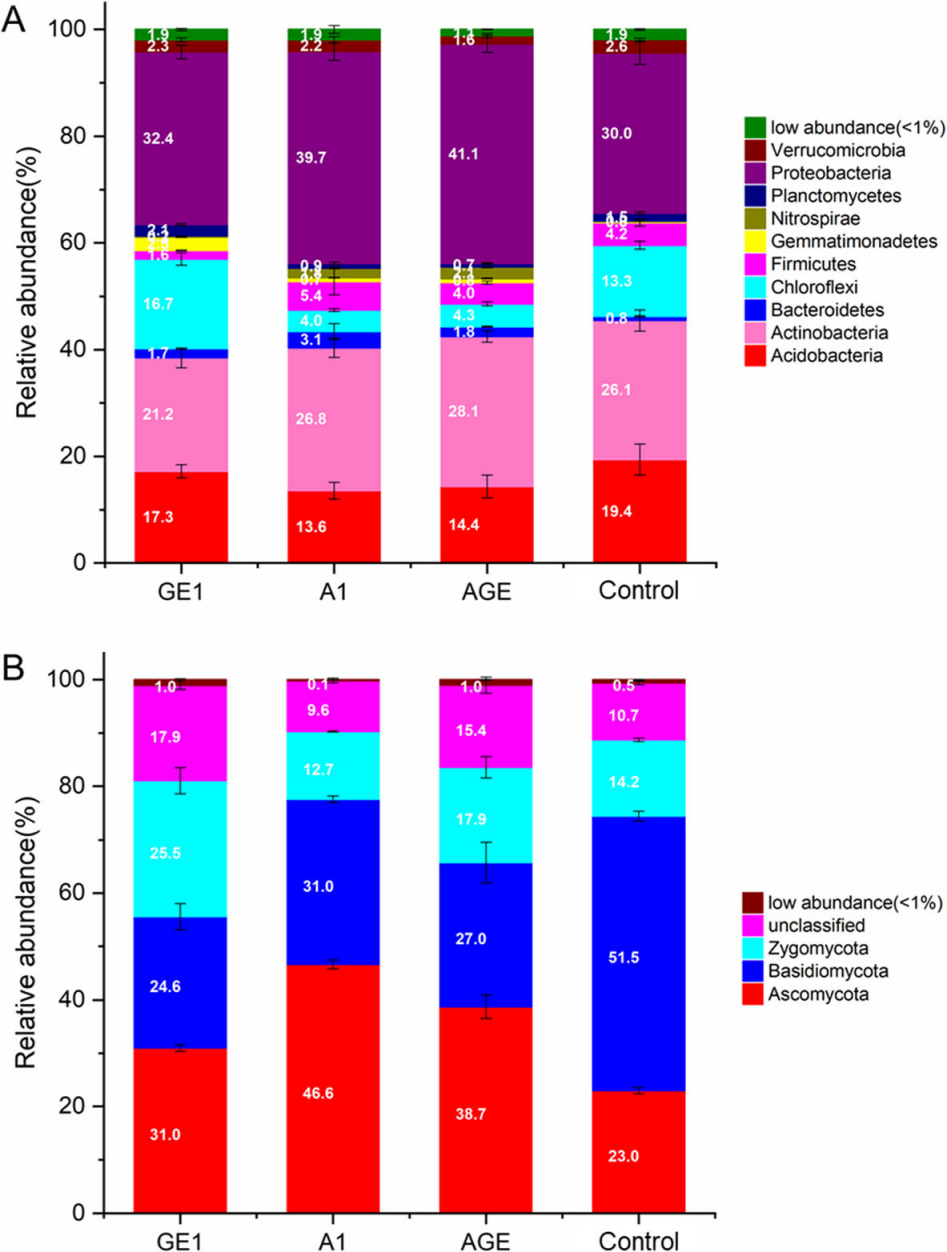
The bacterial community composition (**a**) and fungal community composition (**b**) at phyla level in different rhizoshere and mycorrhizoshere soil samples. GE1, A1, AGE, and control represent the rhizoshere or mycorrhizoshere soil of *G. elata* tubers, *A. mellea* rhizomorphs, *G. elata* tubers with *A. mellea* rhizomorphs, and unplanted, respectively

**Fig. 5:**
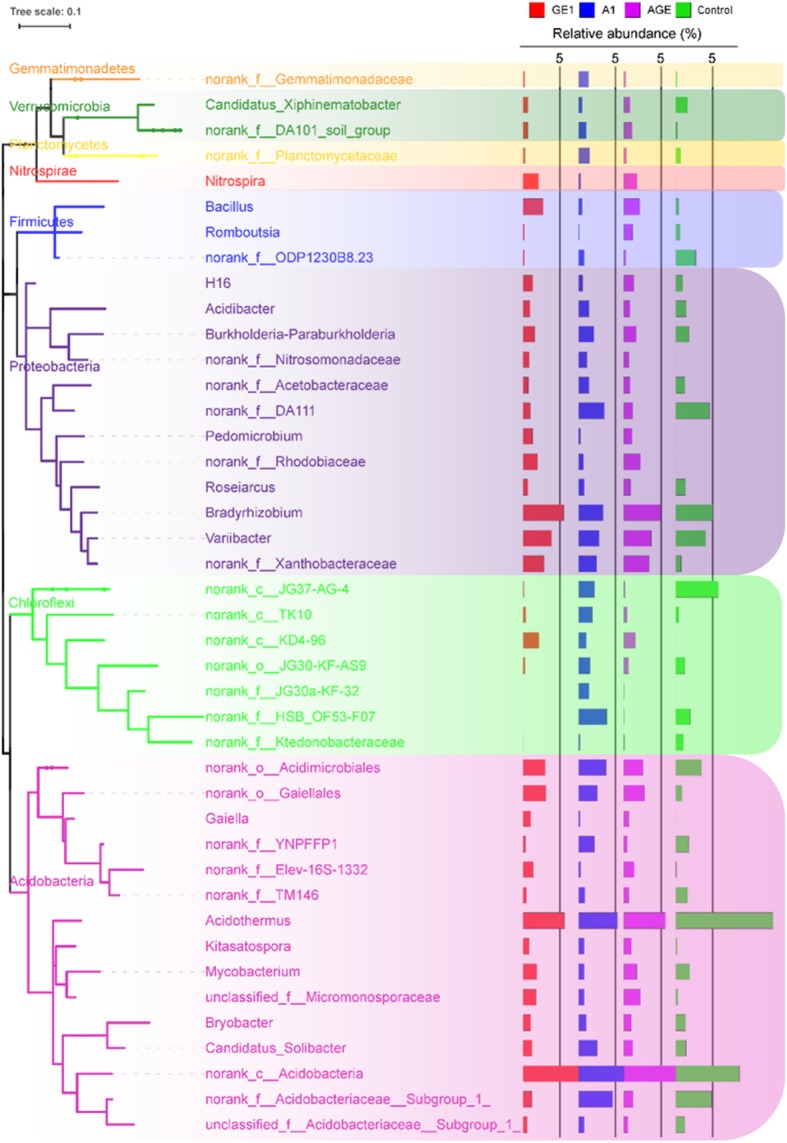
Relative abundance more than 1% bacterial genus in different rhizoshere and mycorrhizoshere soil samples. GE1, A1, AGE, and control represent the rhizoshere or mycorrhizoshere soil of *G. elata* tubers, *A. mellea* rhizomorphs, *G. elata* tubers with *A. mellea* rhizomorphs, and unplanted, respectively

In the total V3V4 rDNA amplicons, almost OTUs (98.9–98.1%) in all soil categories were classified as ten mains phylum, including Verrucomicrobia (1.6–2.6%), Proteobacteria(30.0–41.1%), Planctomycetes (0.7–2.1%), Nitrospirae (0.2–2.1%), Gemmatimonadetes (0.3–2.5%), Firmicutes (0.3–2.5%), Chloroflexi (4.0–16.7%), Bacteroidetes (0.8–3.1%), Actinobacteria (21.2–28.1%), and Acidobacteria (13.6–19.4%) (Fig. 4a). However, obvious variation trends at the phylum level were observed among the A1, GE1, AGE, and control (Fig. 4a). Compared to control, the relative abundance of Firmicutes and Actinobacteria were significantly decreased, while the relative abundance of Chloroflexi, Proteobacteria, Planctomycetes, and Gemmatimonadetes were significantly increased in GE1. However, the variation trends of Firmicutes, Chloroflexi, and Planctomycetes in A1 and AGE were the opposite (Fig. 4a). Furthermore, at the genus level, Genera with the relative abundance greater than 1% were used to evaluate and analyse the bacteria community variation, and itwas found that there was a similar variation trendscompared with the phylum level (Fig. 5).

**Fig. 6:**
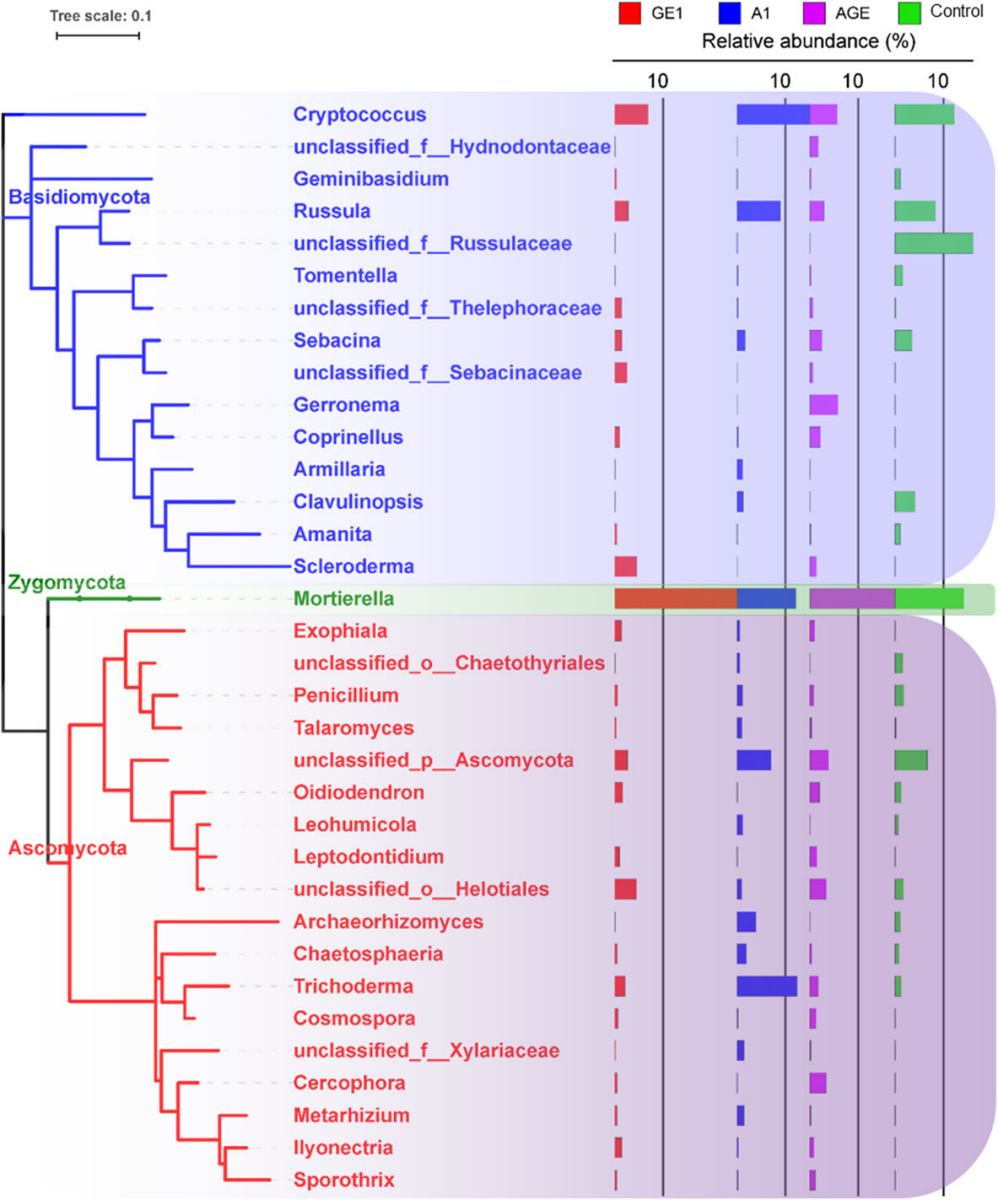
Relative abundance more than 1% fungal genus in different rhizoshere and mycorrhizoshere soil samples. GE1, A1, AGE, and control represent the rhizoshere or mycorrhizoshere soil of *G. elata* tubers, *A. mellea* rhizomorphs, *G. elata* tubers with *A. mellea* rhizomorphs, and unplanted, respectively

For the fungal community, the majority OTUs (82.1–90.4%) were classified as three mains phylum including Ascomycota (23.0–46.6%), Basidiomycota (24.6–51.5%), and Zycomycota (12.7–25.5%). Obvious variation trends at the phylum level were also observed among the A1, GE1, AGE, and control (Fig. 4b). The relative abundance of Ascomycota was significantly increased while the relative abundance of Basidiomycota was decreased among A1, GE1, and AGE under *G. elata* cropping process. But, compared to control, the relative abundance of Zycomycota was increased in GE1 and AGE while decreased in A1 under *G. elata* cropping process (Fig. 4b). Furthermore, at the genus level, Genera with the relative abundance greater than 1% were used to evaluate and analyse the fungus community variation. The relative abundance of Trichoderma, Archaeorhizomyces, Metarhizium, Penicillium, Talaromyces of Ascomycota were significantly increased, while the relative abundance of Coprinellus, Amanita, Scleroderma of Basidiomycota were significantly decreased in A1 compared with control (Fig. 6).

### Network analysis of microbiome shifting patterns under *G. elata* cropping process

**Fig. 7:**
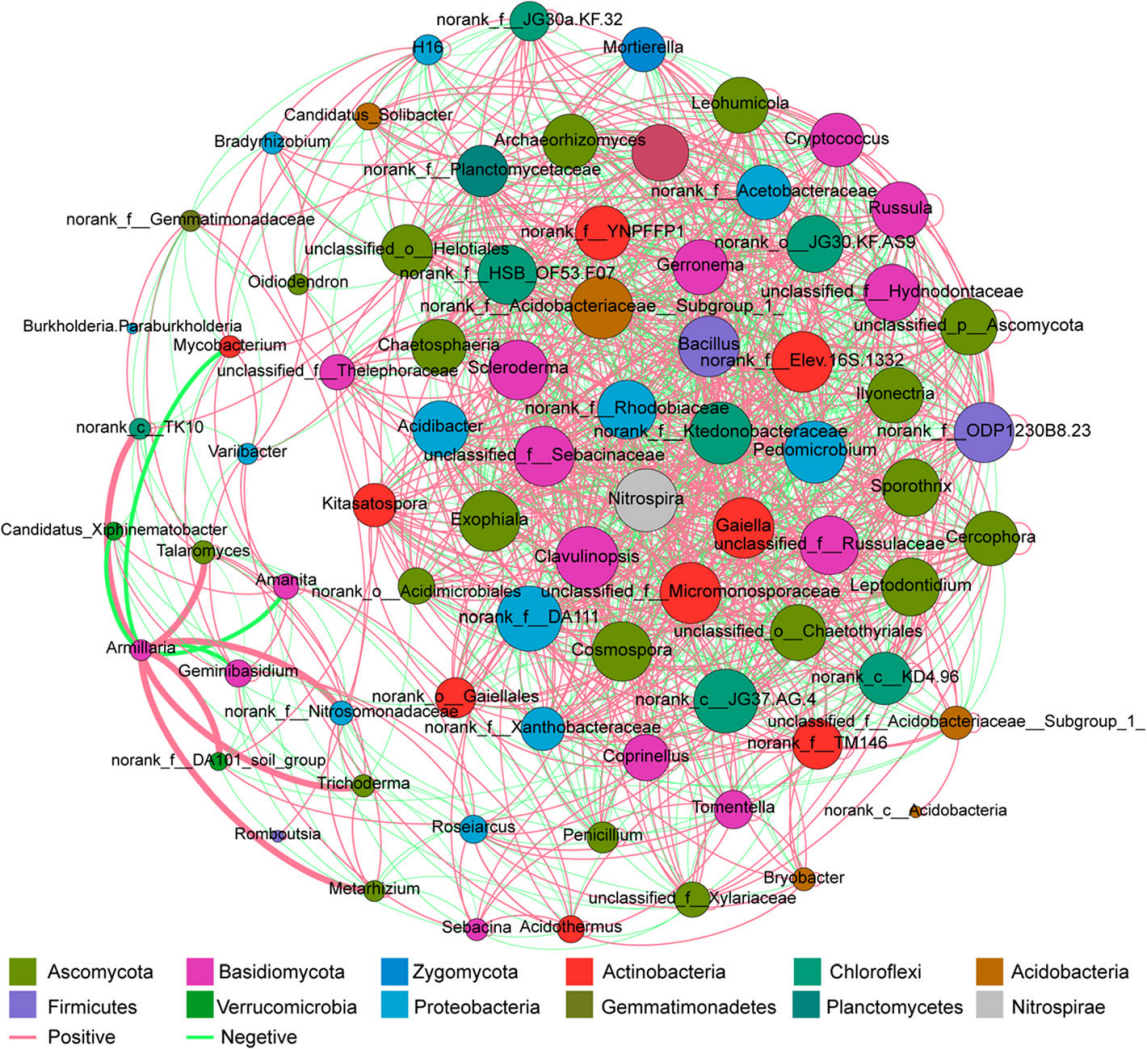
Co-occurrence analysis revealed that microbial community composition reshaped under agriculture process. GE1, A1, AGE, and control represent the rhizoshere or mycorrhizoshere soil of *G. elata* tubers, *A. mellea* rhizomorphs, *G. elata* tubers with *A. mellea* rhizomorphs, and unplanted, respectively

A network composed of 76 nodes and 1314 edges was constructed to describe the complex relationships among soil microbiota in *G. elata* cropping system (Fig. 7). The network average path length was 1.77 and the network diameter was 5.00. All 76 nodes were assigned to 9 bacterial phyla and 3 fungal phyla. Eight phyla, Ascomycota (26.48%), Basidiomycota (19.48%), Proteobacteria (15.58%), Actinobacteria (11.69%), Chloroflexi (9.09%), Acidobacteria (6.49%), Firmicutes (3.90%), and Verrucomicrobia (2.60%), accounted for 93.51% of all OTUs. The 76 genera were significant correlated with *G. elata* cropping process, in which genus *Armillaria* significantly positive interacted with genus *Talaromyces*, *Metarhizium*, and *Trichoderma* (Ascomycota), genus *Mycobacterium* of (Actinabacteria) and genus *Candidatius*-*Xiphinematobacter* (Verrucomicrobia). And more, we found that Ascomycota fungus (19 nodes) had more complex correlation with *G. elata* cropping process than Basidiomycota (13 nodes) (Fig. 7 and S2, Table S3). *Bacillus* interacted with 49 genus including 25 positive interaction genus and 24 negative interaction genus (Fig. 7 and S3, Table S3), which means *Bacillus* had important role to shape the microbial community under the *G. elata* cropping process.

## Discussion

Currently, a battery of studies showed that lots of plants including crops and medicinal plants could shape the microbial community under agriculture process. This study is the first to use culture-independent next generation sequencing techniques to demonstrate the microbial community associated with *G. elata* and *A. mellea* under ternary planting system. Like many researchers reported in wheat [2], potato [27], and *R. glutinosa* [6], various kinds of microbe floras including fungus of the phyla Basidiomycota and Ascomycota and bacteria of the phyla Proteobacteria, Firmicutes, Chloroflexi, Actinobacteria, Acidobacteria, were significantly impacted by *G. elata* and *A. mellea* under agriculture process (Fig. 4).

Highly diverse microbial community is the important index for soil health [28], which is structurally and functionally influenced by plant and soil type [35]. As well as microbial community also can influence the plant growth, resistance to pathogens, and tolerance to stress with feedback [12]. Many previous studies showed that microbial community diversity were daftly reduced in rhizosphere soil under banana [28] and *P. notoginseng* [36] agriculture process. In contrast, alpha diversity analysis showed that the diversity of soil microbial communities in *A. mellea* mycorrhizosphere and *G. elata* rhizosphere were significantly increased after *G. elata* cropping process (Fig. 2). The reason for this contrary phenomenon may be that *A. mellea* as a mycorrhiza fungi can promote the growth of *G. elata* and increase the organic matter in mycorhizosphere soil through decaying wood materials in the ternary agriculture system [28]. Interestingly, by comparing the mycorrhizosphere soil of *A. mellea*, we found that *G. elata* could reduce the richness and diversity of bacterial community in rhizosphere of *G. elata*, but increase the richness and diversity of fungal community in rhizosphere of *G. elata* (Fig. 2). This results is consistent with the previously studies, in which *Rehmannia glutinosa* resulted in a reduction in the richness and diversity of beneficial bacteria in the monoculture regime [6], and continuous cropping of vanilla resulted in an increment in the richness and diversity of fungal pathogens [37]. The phenomenon can be explained by the selective effect of *G. elata* on microbe flora in rhizosphere soil, but its mechanism needs further study.

Based on the analysis of microbial community composition at the phyla level,it was found that compared with control, both *G. elata* and *A. mellea* significantly decreased the relative abundance of Basidiomycete and increased the relative abundance of Ascomycete (Fig. 4). This result is in line with our study that fermentation broth of *A. mellea* significantly inhibited the growth of *Phallus impudicus* (data not show). Furthermore,compared with A1, GE1 and AGE significantly decreased the relative abundance of Ascomycete (genus *Penicillium*, *Talaromyces*, *Leohumicola*, *Archaeorhizomyces*, *Chaetoshpaeria*, *Metarhizium*, and *Trichoderma*) and Basidiomycetes (genus *Cryptococcus*, *Russula*, and *Clavulinopisis*) (Figs. 4, 5). This may be due to the fact that *G. elata* can produce antifungal proteins with strong inhibitory effects on a variety of fungus. For examples, Hu and Huang’s research showed that gatrodia antifungal protein (GAP) or gastrodianin produced by *G. elata* had strongly antifungal activity against *Trichoderma virde*, *Fusarium oxysporum* and *Pyricularia oryzae* etc. [38]. All these results strongly indicated that the shift pattern of soil microbial communities composition in rhizosphere of *G. elata* tuber under agriculture process are similar to those of other medicinal plants such as *P. heterophylla* [3, 4] and *R. glutinous* [5, 6], but in mycorrhizosphere of *A. mellea* rhizomorphs is completely different.

Taxonomic analysis showed that the relative abundance of ectomycorrhizal fungus *Russula*, *Sebacina*, and *Amanita* decreased in mycorrhizosphere soil of *A. mellea* rhizomorphs, except *Scleroderma* (Fig. 6). This is similar to *Tuber borchii* as a ectomycorrhizal fungi, which significantly reduced the abundance of other competitive mycorrhizal fungi in the early symbiotic stage with *Corylus avellana* [39]. On the other hand, the relative abundance of ectomycorrhizal fungus *Russula*, *Sebacina*, and *Amanita* also decreased in rhizosphere soil of *G. elata* tube, except *Scleroderma* (Fig. 6). Many studies have reported that ectomycorrhizal fungi such as *Sebacina* are significantly regulated by their host [40]. Oh et al. demonstrated that the abundance of genus *Russula*, *Sebacina*, and *Amanita* were highly influenced by the host plant *Querus mongolica* and their abundance in soil was higher than in roots of *Q. mongolica* [41]. Similarly, Maghnia et al. pointed out that the change of genus *Russula* was correlated with host *Querus suber* in their Northern Moroccan forest [42]. But the mechanism of how *G. elata* and *A. mellea* reduce the mycorrhizal fungi composition in (mycor) rhizoshpere needs further study.

Besides, it was found that saprophytes and pathogens such as *Exophiala*, *Leptodontidium*, *Cosmospora*, *Cercophora*, *Metarhizium*, *Ilyonectria*, and *Sporothrix*, were enriched in rhizoshere of *G. elata* and in mycorrhizosphere of *A. mellea* through taxonomic analysis (Fig. 6). This is in consistent with previous study that the fungus of *Ilyonectria*, a pathogen of Rusty Roots of *Panax ginseng*, was enriched in rhizosphere soil of Diseased-roots [36]. Similarly, Wang et al’s research showed that the relative abundance of *Ilyonectria* in different depths apple orchard soil was increased by the application of bioorganic fertilizer through the high-throughput sequencing [43]. But, the enrichment degree of these pathogens in mycorrhizosphere of *A. mellea* was lower than that in rhizoshere of *G. elata*. This result demonstrated that *A. mellea* can reduce the enrichment of pathogens in mycorrhizosphere soil. It is coincide with *Tuber borchii* as ectomycorrhizal fungi similar with *A. mellea*, significantly decreased the abundance of pathogenic fungi during early symbiotic stage with *Corylus avellana* [39]. However, the mechanism of different effects of *A. mellea* and *G. elata* on the saprophytes and pathogens composition in rhizoshphere needs further study.

There were research showed that *Nitrospira* as a kinds of chemolithoautotrophic microorganisms have the ability to oxidate ammonia via nitrite to nitrate [44, 45]. And more, Yuan el al found that rhizomes of *G. elata* produced large amounts of ammonium by urease hydrolysis of urea [25]. Interestingly, the analysis of bacterial community composition at genus level showed that the relative abundance of *Nitrospira*, *Bacillus*, *Pedomicrobium*, norank_f_Xanthobacteraceae, and *Gaiella* in GE1 and AGE were higher than that in A1 and control (Fig. 6). This results showed that rhizomes of *G. elata* may specifically attract high nitrogen-depend bacteria such as *Nitrospira*, *Bacillus*, *Pedomicrobium*, norank_f_Xanthobacteraceae, and *Gaiella* in its rhizosphere soil though ammonium production.

## Conclusions

**Fig. 8:**
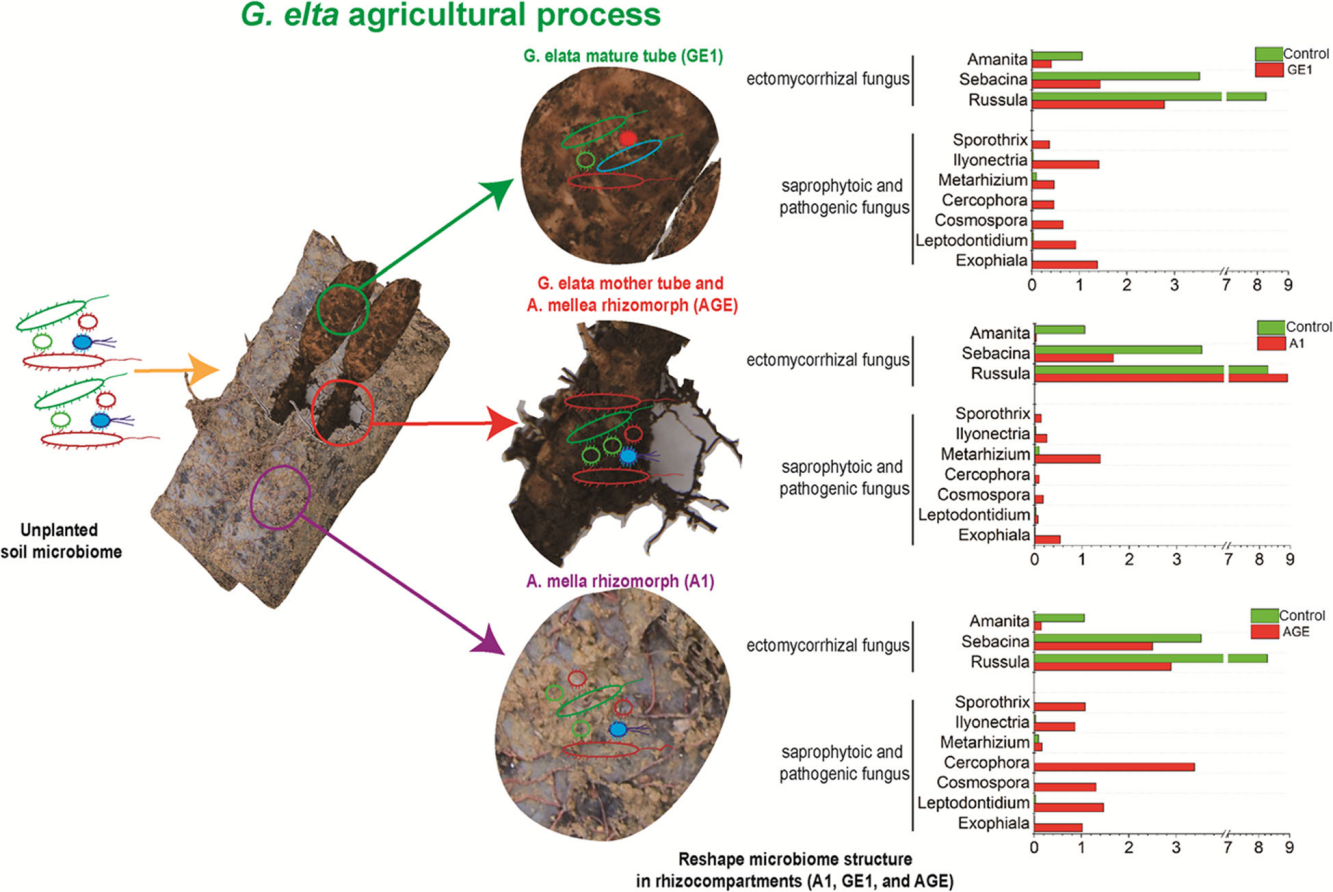
Schematic representation of the proposed interactions between root-associated microbe floras with *G. elata* and *A. mellea* under agriculture process. GE1, A1, AGE, and control represent the rhizoshere or mycorrhizoshere soil of *G. elata* tubers, *A. mellea* rhizomorphs, *G. elata* tubers with *A. mellea* rhizomorphs, and unplanted, respectively

Our results showed that *G. elata* agriculture process clearly affected the root-associated microbial communities in both the rhizosphere and mycorrhizosphere soil. It was found that the richness and diversity of microbial communities in unplanted soil significantly increased under cropping process. In addition, the relative abundance of predominant taxa in mycorrhizosphere of *A. mellea* was opposite in rhizosphere of *G. elata*, including the fungal phyla (i.e., Zygomycota, Basidiomycota, and Ascomycota) and the bacterial phyla (i.e., Proteobacteria, Nitrospirae, Firmicutes, Chloroflexi, Bacteroidetes, Actinobacteria, and Acidobacteria). In particular, several members of saprophytoic and pathogenic fungus (i.e., *Exophiala*, *Leptodontidium*, *Cosmospora*, *Cercophora*, *Metarhizium*, *Ilyonectria*, and *Sporothrix*) were significantly enriched in mycorrhizosphere soil of *A. mellea* and rhizoshpere soil of *G. elata*, while beneficial ectomycorrhizal fungus (i.e., *Russula*, *Sebacina*, and *Amanita*) were significantly reduced in rhizosphere soil (Fig. 8). Further studies on maintaining the balance of soil microbiome in *A. mellea* mycorrhizosphere and *G. elata* rhizosphere under agriculture may provide a promising avenue for high yield and high quality cultivation of *G. elata*.

## Methods

### Planting and sampling of Rhizosphere soil of different Partment in *G. elata* cropping system

The cultivation location of *G. elata* was located at Dafang County, Guizhou Province, China (N27°10′29″, E105°56′7″) and is carried out according to WHO guidelines on good agricultural and collection practices (GACP) for medicinal plants (https://www.who.int/medicines/publications/traditional/gacp2004/en/). The mean annual temperature and precipitation in this area are 11.8 °C and 1150 mm, respectively. The *G. elata* Blume were widely planted on a large-scale in the geo-authentic areas and were selected as the experimental material. The experiment was performed in a single field site that it was newly reclaimed land and ensure the uniform growth condition among repetitions. The *G. elata* was cultivated as follow: first, holes with the size of 100 cm (length) × 30 cm (width) × 30–50 cm (depth) for each, were dug in the field; next, 5 pieces of chestnut wood (Ф = 5–10 cm, length = 30 cm) fully infected by *A. mella* were put parallel on the bottom of each hole; then, 1 kg juvenile tubers of *G. elata* were put evenly on the chestnut wood, and two vials (ca. 1 L in total) of *A. mella*-infected twigs and leaves were crushed and sprinkled on the surface of the juvenile tubes and chestnut wood; finally, soils were filled back into the holes and covered with straw or leaves. *G. elata* was planted on November 12th, 2017 and sampled the rhizosphere and mycorrhizosphere soil on November 7th, 2018. *G. elata* of all experiments was identified by Professor Weike Jiang, and its voucher specimen was deposit on National Specimen Information Infrastructure (http://www.papc.cn/html/folder/1-1.htm) as deposition number 000031805.

Soils from each rhizocompartment in *G. elata* ternary cropping system were collected (We got the permission from Guizhou Wumeng Fungi Industry Co., Ltd.), using a modification of the method previously described by [6]. In brief, *G. elata* mature tuber (GE1), *G. elata* mother tuber contained with rhizomorph of *A. mellea* (AGE), and rhizomorph of *A. mellea* (A1) were carefully uprooted and shaken to remove loosely attached soil on the GE1, AGE and A1. The GE1, AGE and A1 with tightly attached soil were added into a 50 mL centrifuge tube with 20 mL sterile water. After vortex mixing at 200 rpm for 20 min, the washed tubers and rhizomorph of *A. mellea* were removed and the soil suspension was centrifuged at 11,000 rpm for 10 min. The deposit was kept as the rhizosphere soil. And the unplanted field soil as the control. Each category was collected with four replications. In total, 16 samples were stored at − 70 °C for subsequent DNA extraction.

### DNA extract and ITS and V3V4 fragment amplification

Microbial DNA was extracted from 16 samples using the E.Z.N.A.® soil DNA Kit (Omega Bio-tech, Norcross, GA, U.S.) according to manufacturer’s protocols. The final DNA concentration and purification were determined by NanoDrop 2000 UV-vis spectrophotometer (Thermo Scientific, Wilmington, USA), and DNA quality was checked by 1% agarose gel electrophoresis. The V3-V4 hypervariable regions of the bacteria 16S rDNA gene were selected and amplified with primers 338F (5′-ACTCCTACGGGAGGCAGCAG-3′) and 806R (5′-GGACTACHVGGGTWTCTAAT-3′) by thermocycler PCR system (GeneAmp 9700, ABI, USA), as well as ITS1 regions of the fungus rDNA gene were selected and amplified with primers ITS1F (5′-CTTGGTCATTTAGAGGAAGTAA-3′) and ITS2R (5′-GCTGCGTTCTTCATCGATGC-3′). The PCR reactions were conducted by following program: pre-denaturation 3 min at 95 °C, 27 cycles of denaturation 30 s at 95 °C, annealing 30 s at 55 °C, and elongation 45 s at 72 °C, and a final extension at 72 °C for 10 min. PCR reactions were performed in triplicate 20 μL mixture containing 4 μL of 5 × FastPfu Buffer, 2 μL of 2.5 mM dNTPs, 0.8 μL of each primer (5 μM), 0.4 μL of FastPfu Polymerase and 10 ng of template DNA.

### ITS and V3V4 library preparation and Illumina sequencing

PCR products were isolated and extracted from a 2% agarose gel, further purified by using the AxyPrep DNA Gel Extraction Kit (Axygen Biosciences, Union City, CA, USA) and quantified using QuantiFluor™-ST (Promega, USA) according to the manufacturer’s protocol. Purified amplicons were pooled in equimolar and paired-end sequenced (2 × 300) on an Illumina MiSeq platform (Illumina, San Diego, USA) according to the standard protocols by Majorbio Bio-Pharm Technology Co. Ltd. (Shanghai, China).

### Processing of sequencing data

Trimmomatic were used to quality-filtered raw reads, and then FLASH were used to merge the paired-end reads to a tags from the high quality clean reads following criteria described by [46]. Operational taxonomic units (OTUs) were clustered with 97% similarity cutoff using UPARSE software (version 7.1, http://drive5.com/uparse/) with a novel greedy algorithm that performs chimera filtering and OTU clustering simultaneously. The taxonomy of each V3V4 16S rDNA gene sequence was analyzed by Ribosomal Database Project Classifier algorithm (RDP, V.2.2, http://rdp.cme.msu.edu/) against the Silva (SSU123) 16S rDNA database with 70%confidence threshold, as well as the taxonomy of each ITS1 rDNA gene sequence was analyzed by RDP algorithm against the UNITE database (Version6, Vanemuise, Tartu, Estonia).

### Bioinformatics analysis of microbiome sequencing

Alpha diversity indies were used to evaluate the richness and species diversity for each sample, including observed species, Chao1, ACE, and Shannon and Simpson indexes, which were calculated by Quantitative Insights into Microbial Ecology platform (QIIME, v1.80, Twin Cities, MN, USA, 2017) based on the normalized data. Principal coordinate analysis (PCoA) based on weighted UniFrac distances (WUD) method and Hierarchical clustering analysis (HCA) based on the Bray-Curtis distance (BCD) method were performed to investigate beta-diversity patterns between complex microbiota communities. Venn diagrams were constructed to visualize shared and unique OTUs among samples. The phylogenetic trees analysis of the relative abundance more than 1% genus were conducted by Molecular Evolutionary Genetics Analysis platform (MEGA, V7.0.14, https://www.megasoftware.net/) based on the Neighbor-Joining methods. And the compositions and variation of mycrobiota community were calculated in the interactive tree of life platform based on the relative abundance of genus more than 1% (iTOL, https://itol.embl.de/login.cgi?logout=1). The co-occurrence network analysis of the relative abundance more than 1% genus were calculated by the “psych” package in R software (V3.6.2, https://www.r-project.org/) based on the pairwise spearman method [47], and the filter conditions were significance level with cutoff value *p* < 0.05 and correlation coefficient with threshold value r > 0.7, finally, the co-occurrence diagrams were visualized by Gephi software (V0.9.2, https://gephi.org/).

### Statistical analysis of sequencing data

One-way analysis of variance (ANOVA) based on the Tukey’s test method (p < 0.05, *n* = 4) was carried out by SAS software (V 9.4, https://www.sas.com/en_us/) for multiple comparisons of the relative abundance of the microbiota groups and alpha diversity indices.

## Supplementary information

**Additional file 1: Figure S1.** Simposon index of bacterial (A) and fungal (B) communities in different rhizoshere and mycorrhizoshere soil samples.

**Additional file 2: Figure S2.** Co-occurrence analysis revealed that bacteria community composition reshaped under agriculture process.

**Additional file 3: Figure S3.** Co-occurrence analysis revealed that fungi community composition reshaped under agriculture process.

**Additional file 4: Table S1.** Summary for bacteria V3V4 pyrosequencing and assembly.

**Additional file 5: Table S2.** Summary for fungal ITS pyrosequencing and assembly.

**Additional file 6: Table S3.** The relationship of changed microbiome at different compartment rhizoshphere at genus level.

## Data Availability

All data analyzed during this study are included in this published article and its supplementary information files. Additional raw data from sequencing and quality control datasets are available from the corresponding author upon reasonable request.

## Abbreviations

ITS: Internal transcribed spacer
V3V4: The 3rd and 4th variable regions of 16S rDNA gene
NGS: Next generation sequencing
OTUs: Operational taxonomic units
