# Supplementary figures and images for: Insight to shape of soil microbiome during the ternary cropping system of Gastradia elata
Source: BMC Microbiol. 2020 May 5;20:108. doi: 10.1186/s12866-020-01790-y (PMC7201697; doi:10.1186/s12866-020-01790-y)

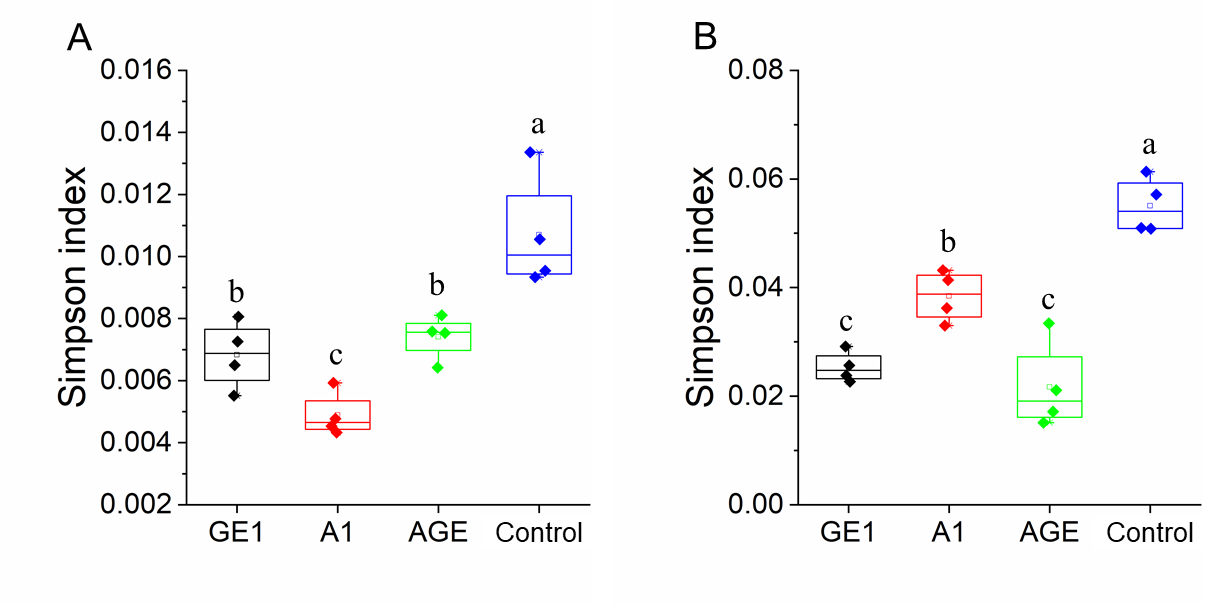

Supplement: Supplementary file 1 — Additional file 1: Figure S1. Simposon index of bacterial (A) and fungal (B) communities in different rhizoshere and mycorrhizoshere soil samples. [file 12866_2020_1790_MOESM1_ESM.png]

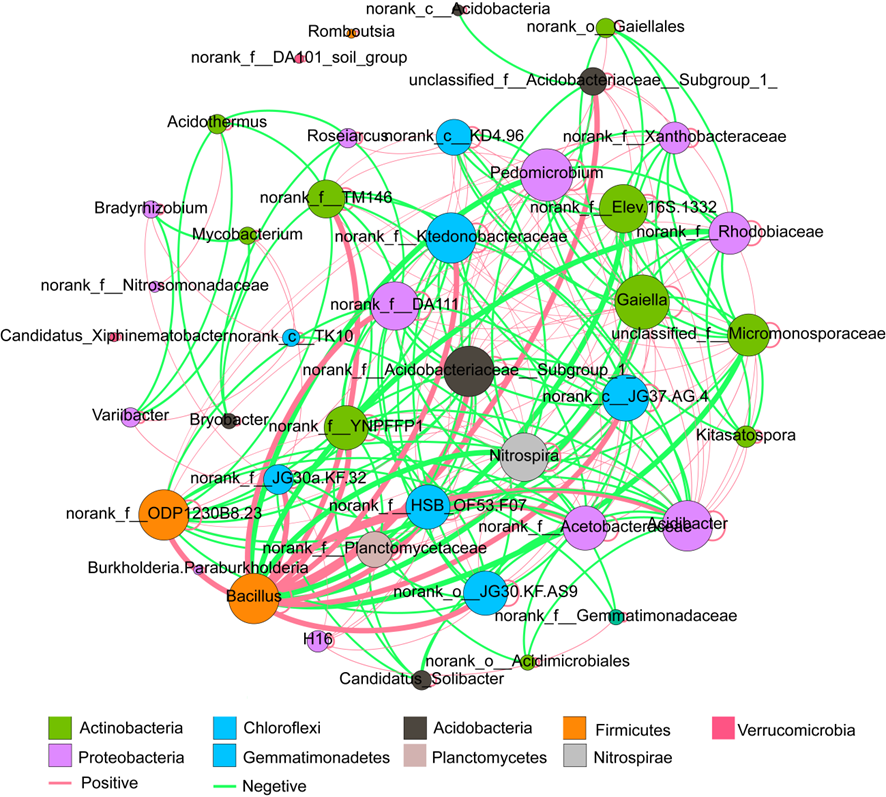

Supplement: Supplementary file 2 — Additional file 2: Figure S2. Co-occurrence analysis revealed that bacteria community composition reshaped under agriculture process. [file 12866_2020_1790_MOESM2_ESM.png]

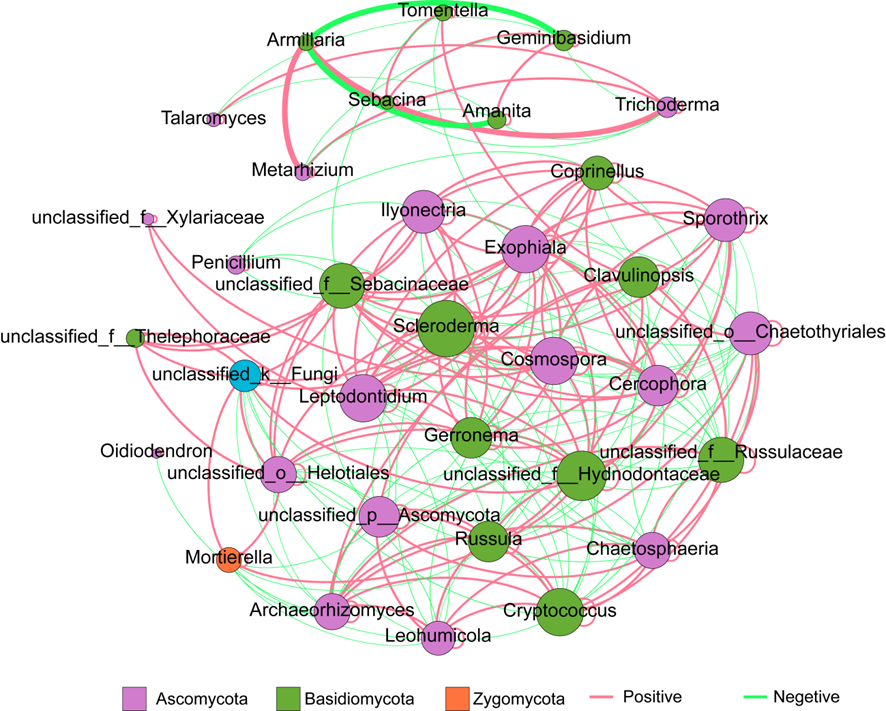

Supplement: Supplementary file 3 — Additional file 3: Figure S3. Co-occurrence analysis revealed that fungi community composition reshaped under agriculture process. [file 12866_2020_1790_MOESM3_ESM.png]
