# Supplementary material for: Insight to shape of soil microbiome during the ternary cropping system of Gastradia elata
Source: BMC Microbiol. 2020 May 5;20:108. doi: 10.1186/s12866-020-01790-y (PMC7201697; doi:10.1186/s12866-020-01790-y)
Supplement: Supplementary file 4 — Additional file 4: Table S1. Summary for bacteria V3V4 pyrosequencing and assembly. [file 12866_2020_1790_MOESM4_ESM.docx]

**Table S1**. Summary for bacteria V3V4 pyrosequencing and assembly

| **Samples** | **Raw Seq No.** | **Effective Seq No.** | **Mean Length (bp)** | **Seq Utilization Ratio (%)** | **OTU No.** |
| --- | --- | --- | --- | --- | --- |
| **GE1_1** | 31892 | 21577 | 435.8140286 | 67.65646557 | 20453 |
| **GE1_2** | 34688 | 23475 | 435.2232472 | 67.67470018 | 23519 |
| **GE1_3** | 30414 | 25041 | 435.8740712 | 82.33379365 | 22092 |
| **GE1_4** | 38101 | 22505 | 435.5628199 | 59.06669116 | 21072 |
| **A1_1** | 37779 | 27340 | 434.2779322 | 72.36824691 | 25799 |
| **A1_2** | 33875 | 26779 | 434.5715129 | 79.05239852 | 25118 |
| **A1_3** | 38555 | 29793 | 434.6426663 | 77.27402412 | 27797 |
| **A1_4** | 41730 | 27435 | 434.1419842 | 65.74406902 | 25758 |
| **ADE_1** | 40803 | 23092 | 434.9550523 | 56.5938779 | 21847 |
| **ADE_2** | 32013 | 19971 | 436.8672414 | 62.38403149 | 16149 |
| **ADE_3** | 33005 | 25964 | 435.2944402 | 78.66686866 | 24412 |
| **ADE_4** | 32114 | 19555 | 434.1823504 | 60.89244566 | 18526 |
| **CK_1** | 38394 | 27304 | 431.8495077 | 71.11527843 | 26366 |
| **CK_2** | 35811 | 25861 | 431.9634191 | 72.21524113 | 24920 |
| **CK_3** | 42466 | 34971 | 432.083714 | 82.35058635 | 33051 |
| **CK_4** | 30673 | 21426 | 432.0756366 | 69.85296515 | 20728 |
| **Average** | 35769.5625 | 25130.5625 | 434.3362265 | 70.32760524 | 23600.4375 |

GE1, A1, AGE, and CK represent the rhizoshere or mycorrhizoshere soil of *G. elata* tubers, *A. mellea* rhizomorphs, *G. elata* tubers with *A. mellea* rhizomorphs, and unplanted, respectively. The numbers followed by the treatments represent the four replicates.
