# Supplementary material for: Insight to shape of soil microbiome during the ternary cropping system of Gastradia elata
Source: BMC Microbiol. 2020 May 5;20:108. doi: 10.1186/s12866-020-01790-y (PMC7201697; doi:10.1186/s12866-020-01790-y)
Supplement: Supplementary file 5 — Additional file 5: Table S2. Summary for fungal ITS pyrosequencing and assembly. [file 12866_2020_1790_MOESM5_ESM.docx]

**Table S2**. Summary for fungal ITS pyrosequencing and assembly

| **Samples** | **Raw Seq Num.** | **Effective Seq Num.** | **Mean Length (bp)** | **Seq Utilization Ratio (%)** | **OTU Num.** |
| --- | --- | --- | --- | --- | --- |
| **GE1_1** | 60867 | 60334 | 261.4648332 | 99.12432024 | 57889 |
| **GE1_2** | 60163 | 59618 | 264.1084554 | 99.09412762 | 57103 |
| **GE1_3** | 49543 | 48999 | 252.545183 | 98.90196395 | 47627 |
| **GE1_4** | 54109 | 53660 | 264.1036426 | 99.1701935 | 51965 |
| **A1_1** | 60424 | 59959 | 257.7493546 | 99.23043824 | 57943 |
| **A1_2** | 60012 | 59581 | 257.1724155 | 99.2818103 | 57539 |
| **A1_3** | 59944 | 67295 | 261.398255 | 112.2631122 | 57452 |
| **A1_4** | 67922 | 59530 | 257.5434322 | 87.64465122 | 64840 |
| **ADE_1** | 60663 | 59819 | 261.2959959 | 98.60870712 | 57004 |
| **ADE_2** | 58574 | 57435 | 259.6150681 | 98.05545122 | 56225 |
| **ADE_3** | 55998 | 55318 | 270.3225294 | 98.78567092 | 53868 |
| **ADE_4** | 58624 | 57719 | 266.7228951 | 98.45626365 | 55105 |
| **CK_1** | 62089 | 61858 | 259.4264524 | 99.62795342 | 61035 |
| **CK_2** | 56524 | 56329 | 259.8471623 | 99.6550138 | 55657 |
| **CK_3** | 61316 | 61070 | 257.9153076 | 99.59879966 | 60063 |
| **CK_4** | 60474 | 60224 | 261.6227304 | 99.5865992 | 59376 |
| **Average** | 59202.875 | 58671.75 | 260.803357 | 99.19281727 | 56918.1875 |

GE1, A1, AGE, and CK represent the rhizoshere or mycorrhizoshere soil of *G. elata* tubers, *A. mellea* rhizomorphs, *G. elata* tubers with *A. mellea* rhizomorphs, and unplanted, respectively. The numbers followed by the treatments represent the four replicates.
